# 基于金属有机框架水凝胶的一步式快速富集检测养殖水体中孔雀石绿

**DOI:** 10.3724/SP.J.1123.2022.04019

**Published:** 2022-08-08

**Authors:** Na LIU, Peiyi LI, Mengmeng SUN, Haiyang QIN, Yuanxin LI, Jincheng LI, Huan LIU, Lidong WU

**Affiliations:** 1.上海海洋大学食品学院, 上海 201306; 1. College of Food Science and Technology, Shanghai Ocean University, Shanghai 201306, China; 2.中国水产科学研究院农业农村部水产品质量安全控制重点实验室, 北京 100141; 2. Key Laboratory of Control of Quality and Safety for Aquatic Products, Ministry of Agriculture and Rural Affairs, Chinese Academy of Fishery Sciences, Beijing 100141, China; 3.大连海洋大学食品科学与工程学院, 辽宁 大连 116023; 3. College of Food Science and Technology, Dalian Ocean University, Dalian 116023, China

**Keywords:** 水凝胶, 金属有机框架物, 海藻酸钠, 孔雀石绿, 吸附, hydrogel, metal-organic framework (MOF), sodium alginate, malachite green, adsorption

## Abstract

孔雀石绿是一种三苯甲烷类化合物,在水产品饲养中对疾病的防治有着不错的疗效,但因对人体健康有危害而被列为禁用药。由于实际样品中成分复杂,对于此类染料的检测方法难以同时兼具富集性好、灵敏度高且方便快速的优点。该工作制备了金属有机框架材料(MOF),采用MOF纳米材料掺杂的水凝胶(PAAM-SA/MOF)对养殖水体中的孔雀石绿进行吸附研究。采用一系列表征手段对MOF、PAAM-SA和PAAM-SA/MOF的微观形貌进行分析,结果表明吸附材料已成功合成。通过优化水凝胶吸附剂用量、吸附时间、孔雀石绿溶液pH、吸附温度、孔雀石绿溶液初始浓度等吸附萃取条件,使溶液中的孔雀石绿基本完全吸附在水凝胶中,在最优条件下,吸附效率最高可达97%。此外,采用不同极性的有机溶剂对吸附的孔雀石绿进行洗脱,通过优化洗脱液体积,脱附率最高达99%。在最佳条件下,该方法在高、中、低3个水平下的样品加标回收试验中回收率达到84.8%~118.1%,相对标准偏差小于5.1%,方法的检出限为0.083 μg/L(*S/N*=3),定量限为0.25 μg/L(*S/N*=10)。该方法简化了前处理过程,结合了MOF和水凝胶这二者各自的优点,添加的MOF材料可以在水凝胶体系中发挥其良好的吸附性,既解决了传统的MOF材料因粒径太小而回收率低的难题,便于吸附后直接提取,同时也解决了纯水凝胶吸附效率较低的问题,整体上提高了吸附效率和可回收性。实际样品测试表明该新型水凝胶吸附材料可用于养殖水体中孔雀石绿的快速萃取和检测,在食品检测领域具有很大潜力。

## Abstract (en)

Malachite green is a triphenylmethane compound, which has a good effect on disease prevention and control in the breeding of aquatic products, but it is a prohibited drug because it is detrimental to human health. Owing to the low content of target malachite green and complex components in the actual sample, simultaneously achieving good enrichment, high sensitivity, convenience, and rapidity detecting is difficult. Metal-organic framework (MOF) has a multidimensional network structure, good stability, and large specific surface area, and has broad application prospects in adsorption. However, the small particle size of MOF materials and the difficulty of recycling hinder their development. The hydrogel has a three-dimensional network structure that can encapsulate the MOF nanomaterials in the network, enhancing adsorption performance and facilitating separation from the adsorbed solution. In this study, MOF materials were prepared, and hydrogel doped with MOF nanomaterials (polyacrylamide-sodium alginate/metal-organic framework, PAAM-SA/MOF) was used to investigate the adsorption of malachite green in aquaculture water. The transmission electron microscopy was used to characterize MOF nanomaterials, scanning electron microscopy was used to examine the morphology and structure of the hydrogels before and after adsorption of malachite green, representing the successful synthesis of adsorbent materials with excellent properties. The mechanical properties of the hydrogels were investigated using a tensile testing machine, with a maximum tensile strain of up to 300%, without breaking and failing to remove when separated from the solution to be tested. The pore size of PAAM-SA/MOF hydrogel is considerably smaller than that of PAAM-SA, which is beneficial to increase the specific surface area of the adsorbent and thus improve the adsorption performance.

A series of optimizations were performed on the adsorption conditions of the hydrogel adsorbent and the optimized conditions were obtained as follows: the amount of adsorbent used was 0.1 g, adsorption time was 5 h, the pH of malachite green solution was 9, the adsorption temperature was 40 ℃, and the initial concentration of malachite green solution was 100 mg/L. Under these conditions the adsorption efficiency could reach up to 97%. Furthermore, the adsorbed malachite green was eluted with organic solvents of various polarities, and the highest desorption efficiency was achieved when acetonitrile with higher polarity was used as the eluent. Simultaneously, the eluent volume was optimized, with 2 mL acetonitrile added to the malachite green-enriched hydrogel adsorbent to achieve the highest desorption efficiency of 99%. The enriched sample was separated using a Dionex Bonded Silica Products C_18_ column (50 mm×2.1 mm, 3 μm), and eluted with an ammonium acetate-acetonitrile solvent system. The results showed that the limit of detection (LOD, *S/N*=3) was 0.083 μg/L, the limit of quantification (LOQ, *S/N*=10) was 0.25 μg/L, and the spiked recoveries of malachite green at high, medium, and low levels were 84.8%-118.1% with the relative standard deviations less than 5.1%. The pretreatment is simplified using this approach and combines the respective advantages of MOF and hydrogel to enable one-step enrichment of malachite green in aquaculture water. The additional MOF material can exert good adsorption in the hydrogel system, which solves the problem of low recovery of traditional MOF materials caused by tiny particle size, facilitating direct extraction after adsorption, and also solves the problem of low adsorption efficiency of pure hydrogel, improving the overall adsorption efficiency and recyclability. The actual sample test shows that the new hydrogel adsorption material can be used to extract and detect trace malachite green in aquaculture water. It is a novel, fast and convenient pretreatment approach with great potential in food detection.

随着国家工业化的发展和生产力的提高,环境污染成为亟待解决的问题^[1]^,尤其是染料和重金属污染^[2]^,不仅会致癌致畸致突变,甚至会直接威胁到生命^[3,4]^。孔雀石绿(malachite green, MG)是一种常用染料^[5]^,也曾是水产品中的一种抗菌药物,其“三致”效应严重威胁人体健康^[6]^。虽然MG已经被农业农村部明令禁止使用,但仍有不法商家在使用。目前,吸附法、物理氧化法、生物降解法等很多技术被用来处理污染废水^[7]^,吸附法因其操作流程较为简便、成本较低而成为废水中染料等污染物去除的一种重要方法^[8,9]^。可用于吸附孔雀石绿的材料有活性炭、磁性吸附剂、农业废料等^[10]^,但存在吸附效率低、成本高、制备工艺复杂等缺点,且需要施加外力(如离心、过滤、磁铁吸引等)才可与吸附完成后的孔雀石绿溶液分离,不能满足快速检测的需要。

水凝胶是一种具有三维网络状结构的高分子材料^[11]^,通常以海藻酸钠^[12]^、纤维素^[13]^、明胶^[14]^、淀粉^[15]^、透明质酸、壳聚糖等多糖分子作为其中的一种网络结构,与丙烯酰胺、丙烯酸、*N*,*N*-亚甲基双丙烯酰胺等第二种网络结构形成复合的双网络结构水凝胶^[16]^。水凝胶含水量极高,具有很强的亲水性却不会溶于水,因此在固液分离方面具有十分大的应用潜力。研究发现以聚丙烯酰胺-海藻酸钠(PAAM-SA)体系的水凝胶作为吸附剂具有很好的吸附效果^[17]^,主要原因是海藻酸钠中存在许多羟基和羧基,易吸附阳离子染料^[18,19]^。

但是,只有水凝胶作为吸附剂其吸附效果并不能达到最佳^[20]^,吸附率只能达到60%左右,因此我们考虑将多孔的金属有机框架(MOF)材料与水凝胶结合,制备成一种新的吸附剂。粉末状的MOF材料通常吸附后难以与溶液相分离^[21,22]^,因此在吸附领域的发展有所限制^[23,24]^。故我们将水凝胶与MOF这两种优异的吸附材料进行复合,制备成一种便携、可在待测溶液中原位提取且节省时间和经济成本的水凝胶吸附剂。本研究中,对MOF及PAAM-SA、聚丙烯酰胺-海藻酸钠/金属有机框架材料(PAAM-SA/MOF)水凝胶的形貌结构采用多种表征手段进行分析。通过对吸附和脱附条件进行一系列优化,水凝胶吸附剂的吸附效率最高可达97%,脱附效率达99%。对养殖水体中的MG进行了吸附提取,HPLC-MS/MS检测,结果表明该前处理方法大大简化了检测过程,是一种很有潜力的新型吸附剂。

## 1 实验部分

### 1.1 仪器、试剂与材料

F15-8x50cy高速冷冻离心机(赛默飞世尔科技(中国)有限公司), FE28 pH计(梅特勒-托利多仪器(上海)有限公司), SPECORD PLUS 210紫外可见分光光度计(德国耶拿公司), ZEISS Sigma 500场发射扫描电子显微镜(德国蔡司公司), HT7700透射电子显微镜(日本Hitachi公司), X0-18S真空冷冻干燥机(南京先欧仪器制造有限公司), ZQ-990LB万能拉力试验机(东莞市智取精密仪器有限公司), AB SCIEX QTRAP 5500三重四极杆质谱仪(美国AB SCIEX公司)。

海藻酸钠(纯度99%)、 *N*,*N*,*N'*,*N*'-四甲基乙二胺(TEMED)、丙烯酰胺(AAm,纯度≥99%)、 *N*,*N*'-亚甲基双丙烯酰胺(MBAA,纯度99%)、过硫酸铵(APS,纯度≥98%)和二水硫酸钙(CaSO_4_·2H_2_O, 纯度≥99%)购自美国Sigma公司。苯甲酸(BA,纯度99.5%)购于上海阿拉丁试剂公司;四(4-羧苯基)卟啉(TCPP, 纯度97%)购于日本TCI试剂公司;八水氧氯化锆(ZrOCl_2_·8H_2_O, 纯度99.9%)购于北京Inno Chem科技公司;氨水(NH_3_·H_2_O,分析纯)、*N*,*N*-二甲基甲酰胺(DMF,色谱纯)、乙醇(C_2_H_5_OH,色谱纯)和MG(色谱纯)购自上海麦克林生化有限公司;甲醇、乙腈、正己烷(色谱纯)购于美国Fisher Chemical公司。18.2 MΩ·cm超纯水通过Milli-Q(美国Millipore公司)超纯水仪制得。养殖水体实际样品采自北京房山区五渡鲟鱼养殖基地。

### 1.2 孔雀石绿标准溶液配制及标准曲线制作

先配制100 mg/L的孔雀石绿水溶液,分别取0.25、0.5、0.75、1.0、1.25 mL于5支25 mL的比色管中,并加入超纯水至刻度,反复摇匀,即得1~5 mg/L的孔雀石绿标准溶液。用纯水作为对比,用紫外可见分光光度计在616 nm波长下进行吸光度测试,制作标准曲线,线性方程为*A*=0.0273*C*+0.0272, *r*^2^=0.9992(*A*为吸光度,*C*为孔雀石绿溶液的质量浓度,*r*^2^为相关系数)。后续吸附条件考察中的孔雀石绿含量均由该方程计算得出。

精确称量孔雀石绿粉末1 mg,用超纯水溶解于100 mL容量瓶中定容至刻度,得到0.01 g/L的标准储备液,再用超纯水逐级稀释,得到质量浓度分别为0.25、0.5、1.0、10、20 μg/L的标准溶液。该系列溶液用于制作实际样品测定的标准曲线。

### 1.3 MOF材料的制备

参照文献^[25]^方法进行制备,具体如下:取100 mL DMF加入到200 mL锥形瓶中。称取300 mg ZrOCl_2_·8H_2_O、100 mg TCPP和5.6 g BA,超声1 min使其混合均匀。将混匀的混合溶液转移至油浴锅中,设置恒定反应温度为90 ℃,反应4 h。待反应结束后,将得到的产物以13000 r/min转速进行离心后,用DMF洗涤3次,最终将产物溶于DMF中,并定容至50 mL,得到深紫色MOF溶液。

### 1.4 PAAM-SA/MOF水凝胶的制备

将2 mL 50 g/L SA溶液与3 mL 230 g/L 丙烯酰胺、80 μL 45.64 g/L APS、200 μL 2 g/L MBAA、1 mL 0.01 g/L的MOF溶液混合均匀,加入20 mL注射器中,抽真空60 min以除去气泡,再与300 μL 172.2 g/L CaSO_4_溶液、10 μL TEMED快速混合,迅速注入模具中。将模具放在紫外线照射器下避光交联1 h。将交联好的水凝胶用蒸馏水冲洗3次,除去未反应的物质。清洗后的水凝胶放入70 ℃烘箱烘干至恒重,备用。

### 1.5 样品前处理

取0.1 g干燥水凝胶作为吸附剂,加入到含有5 mL待测样品的离心管中,用氢氧化钠溶液调节pH至9,在温度40 ℃下吸附萃取5 h,使目标分析物保留在吸附剂中。加入2 mL乙腈,使孔雀石绿从水凝胶中脱附下来,收集洗脱液过0.45 μm滤膜,进行HPLC-MS/MS测试。

### 1.6 表征

为了解MOF材料的形貌特征,对其进行了透射电镜表征。为进一步了解MOF材料是否成功掺杂在水凝胶中并确定MOF材料在水凝胶中的相对位置,对PAAM-SA/MOF水凝胶进行了SEM表征。拍摄SEM时需要对水凝胶冷冻干燥后做喷金处理。

### 1.7 水凝胶力学性能测试

水凝胶的机械拉伸性能用万能拉力试验机进行了评估,该仪器配备了1 kg的称重传感器,拉伸测试在室温下进行。水凝胶的尺寸为36 mm×18 mm×2.5 mm,呈哑铃形。拉伸速度保持在100 mm/min。

### 1.8 HPLC-MS/MS条件

色谱柱:Dionex Bonded Silica Products C_18_柱(50 mm×2.1 mm, 3 μm);柱温:35 ℃;流动相A:乙酸铵溶液(0.005 mol/L);流动相B:乙腈。梯度洗脱程序:0~2 min, 30%B; 2~5 min, 30%B~90%B; 5~7 min, 90%B; 7~8 min, 90%B~30%B; 8~9 min, 30%B。流速:0.3 mL/min;进样量:5 μL。

采用电喷雾离子(ESI)源,正离子模式,喷雾电压(IS) 5500 V,离子源温度(TEM) 550 ℃,气帘气(CUR)压力241.325 kPa,雾化气(GS1)压力379.225 kPa,辅助加热气(GS2)压力379.225 kPa。采用多反应监测(MRM)模式采集数据,监测离子:母离子*m/z* 329.1,定量离子*m/z* 313.2 (碰撞能50 eV),定性离子*m/z* 208.1(碰撞能60 eV)。

## 2 结果与讨论

### 2.1 PAAM-SA/MOF复合水凝胶的制备及吸附

图1为工作流程图。首先,将制备好的MOF材料与预凝胶溶液混合,混合均匀后在紫外光(波长365 nm)下照射1 h,形成一块完整的水凝胶。水凝胶为SA和PAAM两种网络形成的双网络结构,MOF均匀分布在水凝胶基质中。将交联完成的水凝胶烘干即完成吸附剂的制备。称取一定干燥的水凝胶,浸泡在适当浓度的孔雀石绿溶液中进行吸附,可以看到水凝胶由紫红色变为深蓝色,孔雀石绿溶液几乎变为无色,即水凝胶将孔雀石绿基本吸附完全。随后用一定量有机溶剂对吸附了孔雀石绿的水凝胶进行洗脱,孔雀石绿溶解在有机溶剂中。

**图1:**
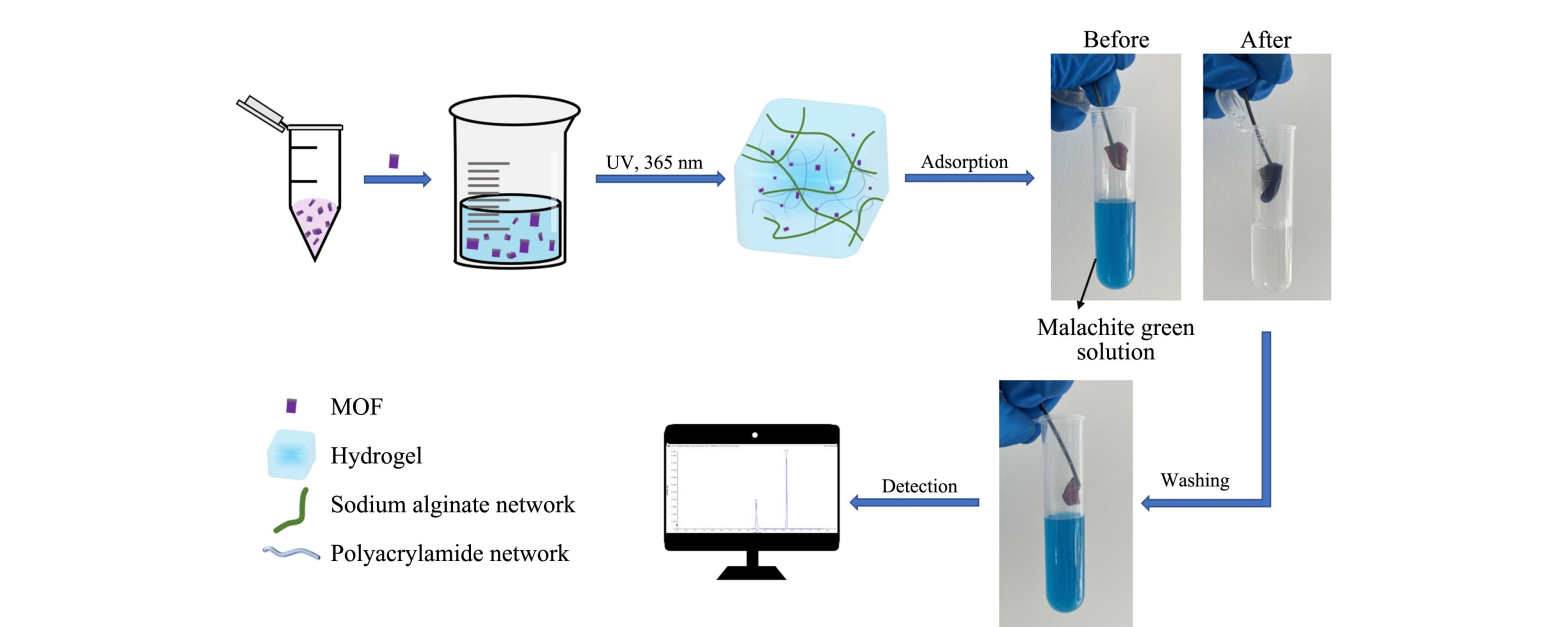
PAAM-SA/MOF水凝胶复合材料吸附及脱附流程图

### 2.2 MOF及水凝胶材料的表征

为明确制备的MOF材料大小及形貌,对其进行透射电镜表征,如图2a、b所示,MOF纳米材料呈现出大小较为均匀的方形。PAAM-SA/MOF复合水凝胶的SEM图见图2c、d,PAAM-SA的SEM图见图2e,可以看出,PAAM-SA的孔径较大,约为16 μm,只能看到水凝胶网络结构中的孔壁,而PAAM-SA/MOF复合水凝胶的孔径有所减小,孔隙增多,表面粗糙,具有许多小孔且内部坚实;从图2f、g可以看出,吸附完孔雀石绿后的水凝胶孔径大大增加,这主要是因为在水溶液中干燥的水凝胶发生了溶胀。吸附后的水凝胶SEM图中能够清晰地看到MOF的立方体结构,既验证了MOF材料均匀掺杂在水凝胶网络结构内,直观地说明本研究成功制备了掺杂有MOF的水凝胶;又表明在加入MOF后,有利于形成水凝胶骨架,增加孔隙,从而能够提高水凝胶对孔雀石绿染料的吸附效果。图2f中可以看到一些除方形结构外的粗糙褶皱,这可能是吸附的孔雀石绿在拍摄SEM时冷冻干燥而析出的晶体。

**图2:**
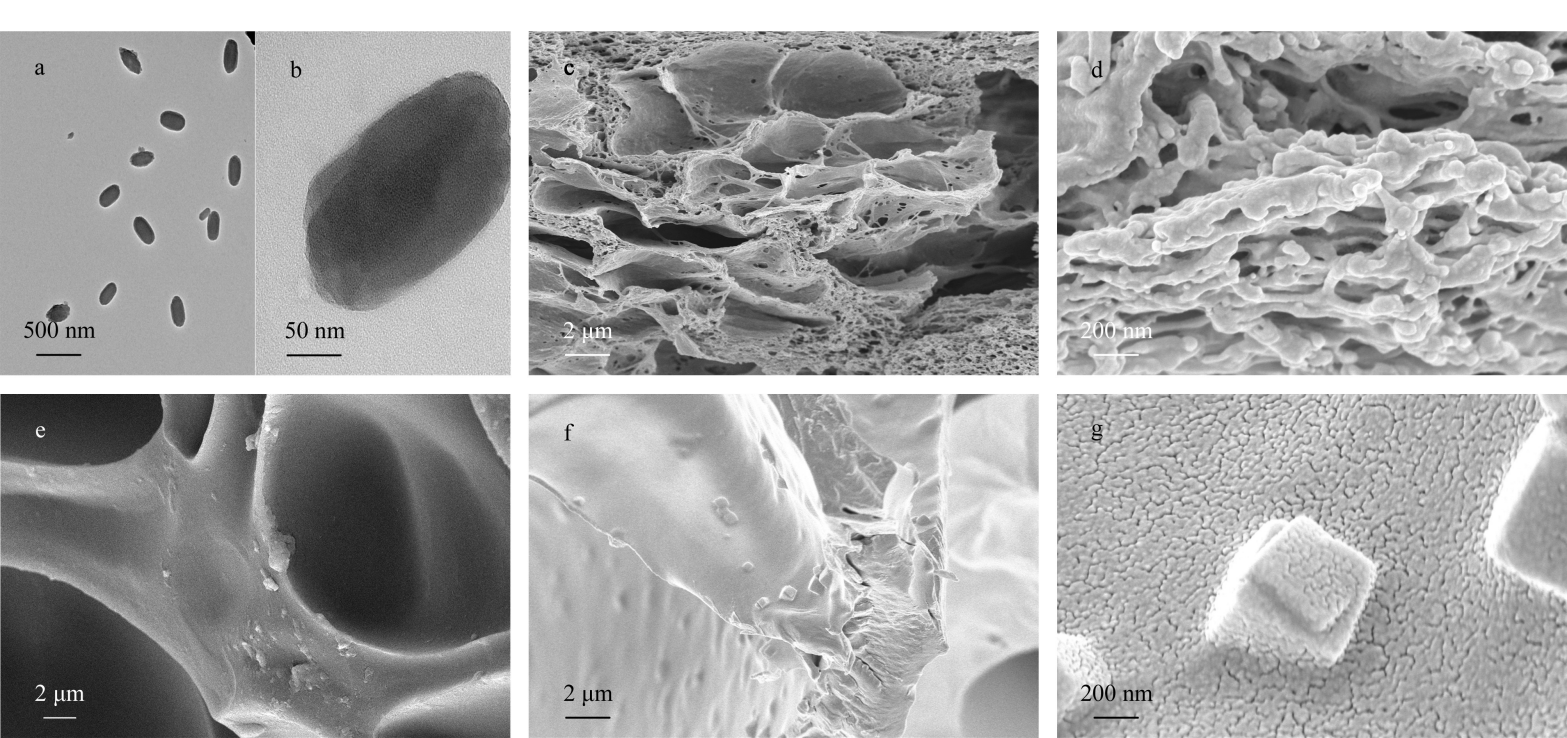
MOF材料的透射电镜图和水凝胶的扫描电镜图

### 2.3 PAAM-SA/MOF水凝胶的力学性能测试

对PAAM-SA/MOF水凝胶进行力学性能测试,实测图和万能拉力试验机测试的应力应变曲线如图3所示。对应图3a拉伸过程,其应力应变曲线如图3b所示,最大应变约为300%,表明制备的水凝胶材料仍然具有一定的力学性能,吸附完成后可以很好地将水凝胶吸附剂与MG溶液分离,不会发生水凝胶破碎而无法分离取出的现象^[26]^。

**图3:**
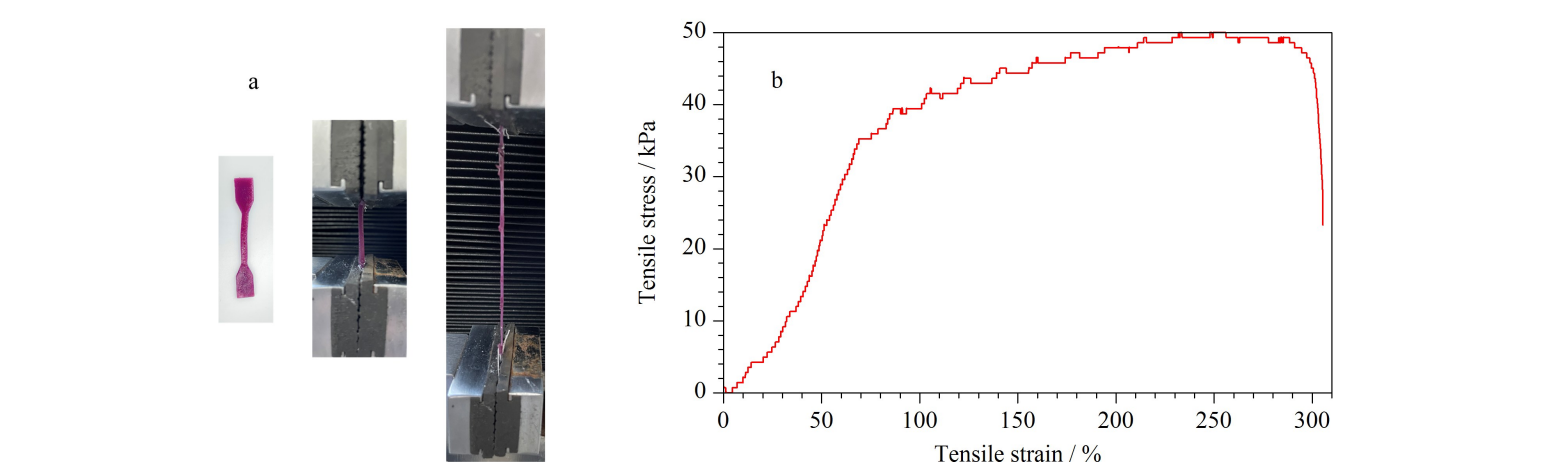
PAAM-SA/MOF水凝胶的力学性能测试

### 2.4 吸附条件的优化

#### 2.4.1 有无MOF对吸附性能的影响

将MOF复合水凝胶PAAM-SA/MOF与不添加MOF材料的纯水凝胶PAAM-SA在同样条件下进行吸附效率的对比。结果如图4a所示,PAAM-SA/MOF水凝胶的吸附率为96.47%,而PAAM-SA水凝胶对MG的吸附率为63.17%,即吸附率比纯水凝胶PAAM-SA增加了约33%。复合水凝胶对MG的吸附效率比纯水凝胶高,表明添加的MOF材料可以很好地在水凝胶体系中发挥良好的吸附作用^[27]^,既解决了传统的MOF材料因粒径太小而回收率低的难题,还解决了纯水凝胶吸附效率较低的问题。

**图4:**
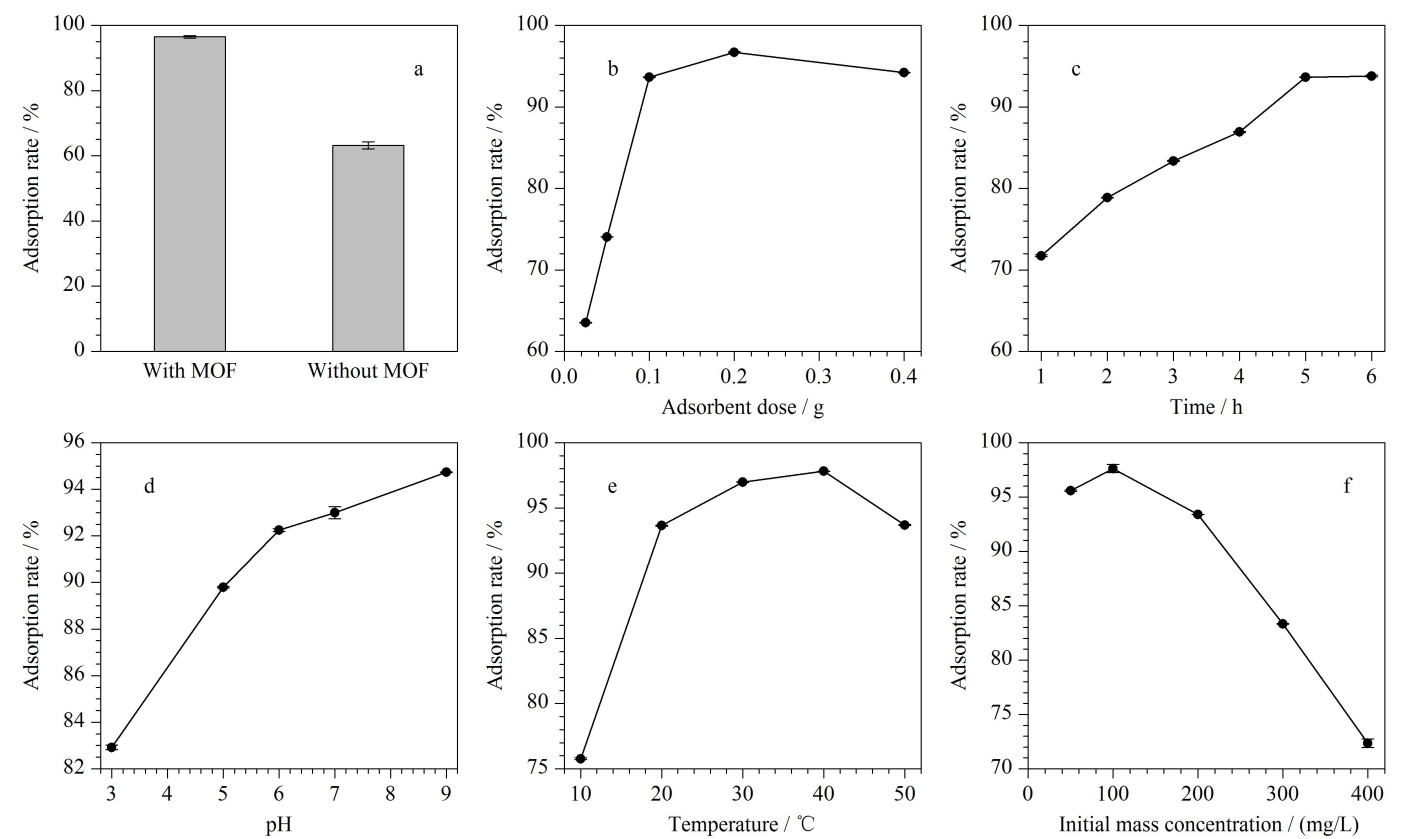
(a)有无MOF材料、(b)水凝胶吸附剂用量、(c)吸附时间、(d)溶液pH、(e)温度和(f)初始MG浓度对吸附效率的影响(n=3)

#### 2.4.2 水凝胶吸附剂用量对吸附性能的影响

干燥水凝胶的用量对于去除效果的影响非常大。结果如图4b所示,当吸附剂质量由0.025 g增加至0.1 g时,吸附率从63%提高到了94%, 0.1 g到0.4 g吸附效率基本保持恒定。考虑到经济成本,确定吸附剂的添加量为0.1 g。

#### 2.4.3 吸附时间对吸附性能的影响

吸附时间的影响见图4c。吸附时间从1 h增加到5 h,吸附率由71%增加到94%之后达到平衡,5 h之后基本达到稳定,因此确定吸附达到平衡的时间为5 h。在一定的时间范围内,对MG的吸附率与吸附作用时间呈正相关,吸附5 h时基本到达饱和点,再增加吸附时间其吸附效率并没有显著提升。因此后续实验都在5 h内完成吸附效率的测试。

#### 2.4.4 pH对吸附性能的影响

图4d是在不同pH条件下,水凝胶对孔雀石绿染料吸附效率的影响。当溶液pH值从3增大至6时,水凝胶对MG的吸附率迅速从83%增大至92%;但当染料溶液pH>6时,水凝胶对MG的吸附率增加缓慢。主要原因是当溶液为酸性条件时,大量的H^+^使吸附剂中的-COO^-^、-NH_2_质子化为-COOH和 ${{{\mathrm{-NH}}_{3}}^{+}}^{\left[28,29\right]}$ ,染料与水凝胶中的羧基、氨基间络合及静电作用降低,且大量的H^+^能与羧基形成分子内氢键^[3]^,使水凝胶溶胀程度降低,吸附效率下降。后续实验控制pH为9进行吸附测试。

#### 2.4.5 温度对吸附性能的影响

温度对水凝胶吸附MG的影响如图4e所示,整体呈现先升高后下降的趋势,吸附温度从10 ℃升高至40 ℃,吸附效率由76%升高到97.5%;高于40 ℃后,吸附效率明显下降。由此可得在适当高的温度下,吸附效率可达到最佳。主要原因是适当的温度下,水凝胶溶胀度增大,更多的MG分子可以与羧基、氨基等官能团构成的吸附位点结合^[30]^,但当温度太高(40~50 ℃)时,可能会降低水凝胶的溶胀度,因此吸附位点减少,降低了吸附效率。综上,选择吸附效果最好的温度40 ℃。

#### 2.4.6 孔雀石绿初始浓度对吸附性能的影响

此外还研究了初始MG浓度对PAAM-SA/MOF吸附MG的影响。PAAM-SA/MOF水凝胶吸附效率随着MG浓度的变化如图4f所示。设置MG初始质量浓度为50、100、200、300、400 mg/L,100 mg/L时吸附效率最高,可达97.62%,而随着MG初始浓度从100 mg/L升高到400 mg/L,吸附效率由97.62%下降到72.36%。主要原因是水凝胶吸附剂的质量一定,当吸附到达饱和点时,剩余的MG分子无法再与水凝胶结合^[31,32]^。

### 2.5 脱附条件的优化

#### 2.5.1 洗脱剂对脱附效率的影响

本实验中采用5种不同的有机溶剂各2 mL对吸附基本达到饱和的5块PAAM-SA/MOF水凝胶在相同环境条件下进行洗脱,选取的有机试剂分别为乙腈、甲醇、乙醇、DMF和正己烷。如图5a所示,极性比较高的乙腈、甲醇对于孔雀石绿的脱附效率较高,用乙腈脱附时达到最高脱附效率,约为99%,可以满足实际工业化处理需要。因此选择乙腈作为较优的洗脱剂来进行后续的洗脱。

**图5:**
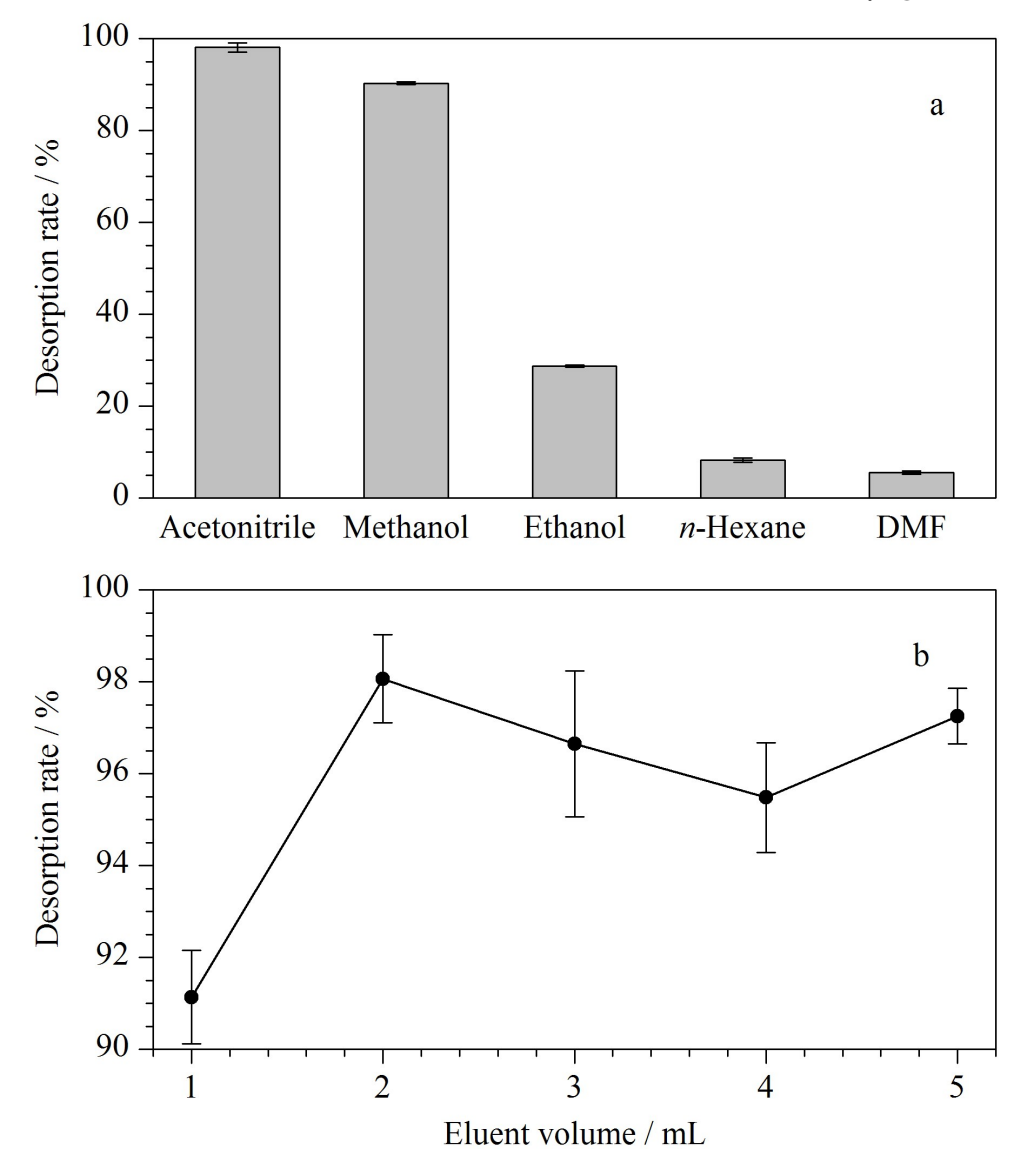
孔雀石绿洗脱条件优化(*n*=3)

#### 2.5.2 洗脱剂体积对脱附效率的影响

洗脱剂的用量对脱附效果也有很大程度影响。若洗脱剂的量太少,吸附的孔雀石绿难以完全脱离;量太多则会增加经济成本,且脱附下来的孔雀石绿浓度可能会太低,不便于后续检测分析。分别用1、2、3、4、5 mL乙腈对吸附条件相同并且吸附达到饱和的水凝胶进行脱附。乙腈体积从1 mL增加到2 mL,脱附效率从91%增加到99%(见图5b)。从2 mL到5 mL时,脱附效率略有下降。同时考虑到经济成本问题,选择用2 mL乙腈来进行脱附处理。

### 2.6 方法学考察

#### 2.6.1 线性关系、检出限和定量限

采用HPLC-MS/MS测定1.2节配制的0.25~20 μg/L标准溶液,结果表明,在0.25~20 μg/L范围内,MG具有良好的线性关系,得到标准曲线方程:*y*=1.74444*x*+0.05728(*x*为质量浓度μg/L, *y*为对应的峰面积),*r*^2^=0.998。以3倍、10倍信噪比计算得出孔雀石绿的检出限为0.083 μg/L,定量限为0.25 μg/L。

#### 2.6.2 准确度与精密度

在空白基质中加入0.5、1.0、5.0 μg/L 3个水平的MG标准溶液以测试该方法的准确度,并计算加标回收率。结果表明养殖水体中孔雀石绿的加标回收率为84.8%~118.1%,相对标准偏差(RSD)为0.12%~5.1%,即该方法满足实际测试中准确度及精密度的要求。

### 2.7 实际样品的分析

将本吸附剂应用在实际样品中进行分析测试,以验证可行性。采集了6个养殖水体样本,经PAAM-SA/MOF水凝胶吸附剂萃取,用1.5节方法对样本中的孔雀石绿进行富集和洗脱,再用HPLC-MS/MS测试,结果均未检出孔雀石绿(典型谱图见图6a)。在养殖水体样本中加标(1.5 μg/L)测试,得到的色谱图如图6b所示,测得的MG含量为1.61 μg/L,该结果表明所制备的新型水凝胶吸附剂可以用于实际样品中低浓度MG的富集,简化前处理过程,且不受样品中其他杂质的干扰,是一种具有潜在应用前景的便携式吸附提取方法。

**图6:**
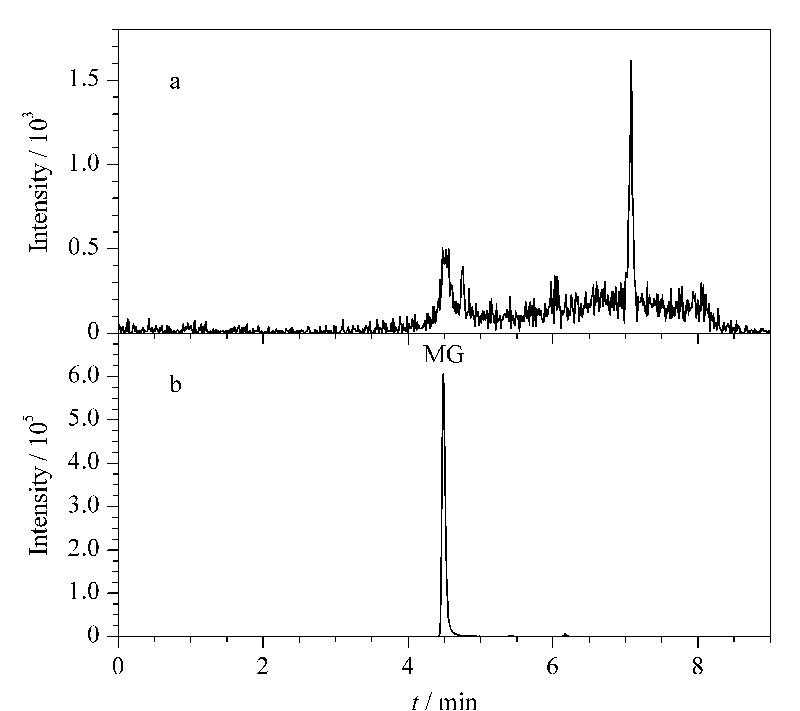
加标(a)前、(b)后养殖水体经MOF复合水凝胶富集的 提取离子色谱图

## 3 结论

本工作成功地将MOF材料包裹在水凝胶中,制备出兼容MOF和水凝胶特点的复合吸附材料,可以更加便携、高效地提取实际样品中的孔雀石绿染料。该研究建立了基于MOF复合水凝胶的快速富集方法,同时结合HPLC-MS/MS可以实现对养殖水体中孔雀石绿的检测,在最佳吸附条件下,水凝胶吸附剂显示出较高的吸附能力,可通过有机溶剂进行完全洗脱,简化富集过程,对准确定量分析实际样品中的孔雀石绿具有潜在的应用价值。
